# Retraction: Leyvraz M. et al. An Assessment of the Potential Impact of Fortification of Staples and Condiments on Micronutrient Intake of Young Children and Women of Reproductive Age in Bangladesh. *Nutrients* 2015, *7*, 9960–9971

**DOI:** 10.3390/nu8090542

**Published:** 2016-09-02

**Authors:** Magali Leyvraz, Arnaud Laillou, Sabuktagin Rahman, Tahmeed Ahmed, Ahmed Shafiqur Rahman, Nurul Alam, Santhia Ireen, Dora Panagides

**Affiliations:** 1 Global Alliance for Improved Nutrition (GAIN), Geneva 1211, Switzerland; dpanagides@gainhealth.org; 2 United Nations Children’s Fund (UNICEF), Phnom Penh 12201, Cambodia; alaillou@unicef.org; 3 International Centre for Diarrhoeal Disease Research (ICDDR), Dhaka 1000, Bangladesh; sabuktagin@icddrb.org (S.R.); tahmeed@icddrb.org (T.A.); ashafiq@icddrb.org (A.S.R.); nalam@icddrb.org (N.A.); santhia@icddrb.org (S.I.)

The published paper [1] contains errors that mean that the resulting analysis is incorrect and the conclusions are no longer supported. We have thus decided to retract the original and republish the paper as [2].

The errors are a result of the fortification level standards for rice being incorrectly interpreted as being in mg/kg instead of mg/100 g [3] and therefore the calculation was done with a level ten-fold lower than actually recommended.

We apologize for any inconvenience caused to readers.
